# Paper-based sensors for rapid detection of virulence factor produced by *Pseudomonas aeruginosa*

**DOI:** 10.1371/journal.pone.0194157

**Published:** 2018-03-22

**Authors:** Fatima AlZahra’a Alatraktchi, Jafar Safaa Noori, Georgi Plamenov Tanev, John Mortensen, Maria Dimaki, Helle Krogh Johansen, Jan Madsen, Søren Molin, Winnie E. Svendsen

**Affiliations:** 1 Department of Micro- and Nanotechnology, Technical University of Denmark, Lyngby, Denmark; 2 Novo Nordisk Foundation Center for Biosustainability, Technical University of Denmark, Lyngby, Denmark; 3 Department of Bioengineering and Biomedicine, Technical University of Denmark, Lyngby, Denmark; 4 IPM – Intelligent Pollutant Monitoring ApS, Lyngby, Denmark; 5 Department of Applied Mathematics and Computer Science, Technical University of Denmark, Lyngby Denmark; 6 Department of Science and Environment, Roskilde University, Roskilde, Denmark; 7 Department of Clinical Microbiology, University Hospital Rigshospitalet, Copenhagen, Denmark; 8 Department of Clinical Medicine, Faculty of Health and Medical Sciences, University of Copenhagen, Copenhagen, Denmark; Institute of Materials Science, GERMANY

## Abstract

Pyocyanin is a toxin produced by *Pseudomonas aeruginosa*. Here we describe a novel paper-based electrochemical sensor for pyocyanin detection, manufactured with a simple and inexpensive approach based on electrode printing on paper. The resulting sensors constitute an effective electrochemical method to quantify pyocyanin in bacterial cultures without the conventional time consuming pretreatment of the samples. The electrochemical properties of the paper-based sensors were evaluated by ferri/ferrocyanide as a redox mediator, and showed reliable sensing performance. The paper-based sensors readily allow for the determination of pyocyanin in bacterial cultures with high reproducibility, achieving a limit of detection of 95 nM and a sensitivity of 4.30 μA/μM in standard culture media. Compared to the similar commercial ceramic based sensors, it is a 2.3-fold enhanced performance. The simple in-house fabrication of sensors for pyocyanin quantification allows researchers to understand *in vitro* adaptation of *P*. *aeruginosa* infections via rapid screenings of bacterial cultures that otherwise are expensive and time-consuming.

## Introduction

*Pseudomonas aeruginosa* is an opportunistic pathogen that rarely infects healthy individuals, but often colonizes immunocompromised patients[1]. This pathogen infects critically-ill hospitalized patients; especially it portends patients with more invasive diseases like patients with cystic fibrosis, AIDS or haematological diseases[2–4]. *P*. *aeruginosa* may develop multi-resistance, which makes it difficult to eradicate with antibiotics[3]. Early identification of the bacterium is therefore important to avoid chronic infection to establish[5]. The presence of *P*. *aeruginosa* is detected by culture in patient samples in order to intervene with antibiotic therapy as early as possible when the bacteria is still susceptible to antibiotics[6].

Pyocyanin is a toxin solely secreted by *P*. *aeruginosa*[7]. *P*. *aeruginosa* produces high quantities of pyocyanin during the early colonization phase in order to establish the infection[8]. Thus, pyocyanin has gained increasing interest among researchers and has been studied to understand the infection development in patients[9]. Until recently, it has only been possible to measure pyocyanin by demanding purification procedures of bacterial cultures followed by cumbersome detection using chromatographic or spectrophotometric techniques[10]. The present available method to extract pyocyanin from samples includes the use of the cancerogenic chemical chloroform that easily evaporates into air[11]. Furthermore, the breakdown products of chloroform in air include highly toxic volatiles such as hydrogen chloride and phosgene[12]. Thus avoiding tedious purification and sample handling steps is favorable.

Recently, studies have demonstrated that pyocyanin can be detected by electrochemical methods without any pretreatment of the bacterial samples[13]. Electrochemical sensors for pyocyanin detection are typically based on a three-electrode configuration. Oxidation at specific pre-determined potentials reveals the presence of pyocyanin[14]. The existing commercial three-electrode sensors that have been tested for pyocyanin detection are relatively costly due to the used materials and fabrication procedures. The customized sensors for pyocyanin detection are either fabricated in expensive cleanrooms or screen-printed on ceramic or glass-based substrates[15,16]. To facilitate screening of large quantities of bacterial samples that are needed for detailed research of infection progression, there is a need for inexpensive sensors that precisely can quantify the concentration of pyocyanin[17].

Paper-based electrochemical sensing is an upcoming field that promises sensitive detection[18]. The low-cost fabrication process, inexpensive materials and disposability of paper, make paper-based sensors a good alternative to conventional electrochemical sensors, as it enables fast and low-cost screening and customized electrode designs[19]. Although paper-based sensors are promising, it has to our knowledge not been demonstrated that pyocyanin can be quantified using paper-based sensors.

In the present work, we demonstrate that paper-based sensors can be used for accurate and sensitive detection of pyocyanin. We have fabricated simple paper-based sensors capable of quantifying the signal directly in bacterial cultures. The pyocyanin signal obtained with the paper-based sensors has been compared to signals obtained with commercially available electrochemical sensors, showing a better performance at a significantly lower cost.

## Materials and methods

### Reagents and materials

Potassium hexacyanoferrate(II)triphydrate (P23289-110G, Sigma-Aldrich, Denmark) and Potassium hexacyanoferrate(III) (31253-250G, Sigma-Aldrich, Denmark) were used to prepare 10 mM ferri/ferro cyanide in phosphate buffered saline (PBS). Pyocyanin (ENZ-53001-C001, Enzo, Denmark) dilution series from 1–40 μM was prepared in the bacterial culture medium lysogeny broth (LB). Electrochemical characterizations were carried out with Emstat 3+ potentiostat from Palmsens (KM Utrecht, The Netherlands). The pyocyanin measurements were conducted by a portable potentiostat (PG851, BioLogic, France). The data analysis was conducted in the software NOVA 1.1. The sensor was designed in SolidWorks 2017. Carbon ink was used for screen printing the electrodes (No. CH-8, Jujo chemicalCo., Ltd.) Spectrophotometer (V-1200, VWR) was used to measure the optical density (OD) in bacterial culture samples.

### Fabrication of paper-based sensors

#### Mask fabrication

The desired sensors consisted of a 3-electrode setup with a working electrode (WE), counter electrode (CE) and a reference electrode (RE) configuration. The paper-based sensor design was based on a commercially available screen printed ceramic electrode (DRP110, Dropsens, Spain). The pattern was cut in 0.05 mm thick plastic foil using a laser cutter (Epilog Laser mini, 30w, USA). The plastic foil-based shadow mask was then used for screen-printing of the electrodes on paper.

#### Screen printing

The photo paper was purchased from (Avery Zweckform, Germany, product No. C2549-100). Carbon Ink (No. CH-8, Jujo chemicalCo., Ltd.). The prepared shadow mask was placed on top of the photo paper and a thin layer of the ink was deposited using manual scraper to create the designed electrodes.

#### Ink curing

**Fig 1:**
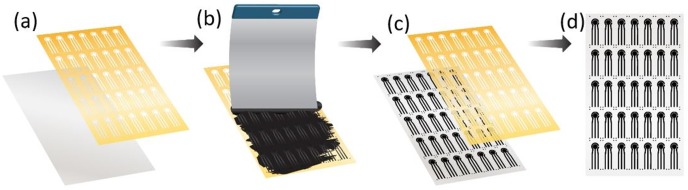
Sensor fabrication process. (a) Placement of the mask on top of the photo paper. (b) Manual scrapping of ink on top of the shadow mask. (c) shadow mask removal. (d) Photo paper with carbon screen printed electrodes.

The paper with the screen printed electrodes was placed in an oven and cured at 120°C for 60 minutes to speed up the annealing of the ink. Finally, the paper was cut out into independent sensors using a scalpel (Fig 1). The small dots served a guide to define the dimensions of each sensor through the cutting process.

The desired patterns were achieved by a reusable shadow-mask containing the electrode patterns. It was possible to fabricate 35 sensors on a regular A6 hard paper. The final sensor dimensions were 33 x 10 x 0.18 mm, the working electrode diameter is 4 mm. Only 0.35 g of ink was used per 35 sensors.

### Electrochemical characterization of paper-based sensors

The paper-based sensors were connected to the potentiostat using a three-pin connector. The paper-based sensors were electrochemically characterized by covering the electrodes with 70 μL ferri/ferrocyanide. Cyclic voltammograms (CVs) was registered at the potential range -1.0 V to 1.0 V. Three cycles were recorded at 10, 50, 100 and 250 mV/s, respectively. The measurements were performed in triplicates. All electrochemical measurements were conducted with respect to the reference electrode.

### Quantification of pyocyanin using paper-based sensors

Square wave voltammetry (SWV) was performed for the pyocyanin dilution series ranging from 1–40 μM. SWVs were performed from -0.7 V to -0.3 V with an amplitude of 0.05 V and a frequency of 10 Hz. The peak height currents at each concentration were considered for the calibration curve. The measurement of each concentration was repeated three times using a fresh sensor for each repetition. The limit of detection was calculated as the concentration corresponding to three times the standard deviation of the lowest detected concentration. The sensitivity was calculated as the slope of the calibration curve.

### Comparing performance to carbon-based and gold based sensors from Dropsens

The performance of commercially available carbon-based (DRP110, Dropsens, Spain) and gold-based screen printed sensors (C220AT, Dropsens, Spain) was compared to the performance of the paper-based sensors by measuring a pyocyanin concentration of 10 μM using SWV.

### Detection of pyocyanin in P. aeruginosa cultures

An overnight culture of the *P*. *aeruginosa* reference strain PAO1 was diluted 1:100 in LB and incubated in 37°C at 250 rpm for 24 hours. The OD at 600 nm (OD600) was measured to quantify the density of the bacteria in each sampled culture. During the 24 hours, 7 samples were collected at different time-points. The pyocyanin concentration was measured using the paper-based sensors and the OD600 was detected to confirm the increasing bacterial number. The electrochemical measurements were repeated three times using a fresh sensor for each recording.

## Results and discussion

### In-house fabrication of sensors

**Fig 2:**
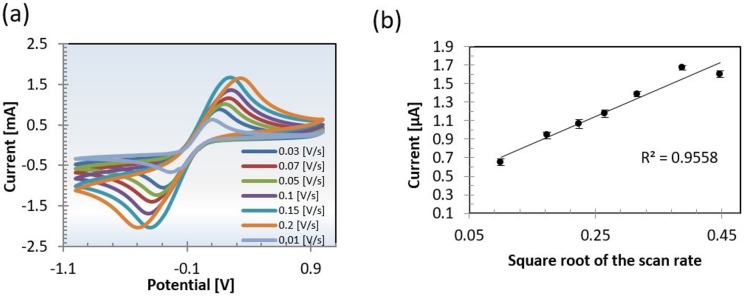
Electrochemical characterization of the paper-based sensors. Evaluation of electrochemical sensor performance by (a) cyclic voltammograms (CV) at various scan rates in ferri/ferrocyanide. (b) The extracted peak currents are linearly proportional with the square roots of the scan rates denoting a quasi-reversible system suitable for analyte quantification. Measurements are conducted against the reference electrode.

Paper-based electrochemical sensors were in-house fabricated by screen-printing carbon ink on paper. The sensing performance of the disposable paper-based sensors was evaluated using CVs of ferri/ferrocyanide at different scan rates (Fig 2a). Ferri/ferrocyanide is widely used for electrochemical characterization of electrode performance. Our measurements showed a linear increase in peak current with increasing square root of scan rate as described in the Randles Sevcik equation for quasi-reversible electron transfer processes (Fig 2b). This behavior is consistent with a semi-infinite diffusion of the redox active species at the electrode, thereby showing the paper-based sensor is suitable for sensing purposes.

### Pyocyanin sensing using paper-based sensors

**Fig 3:**
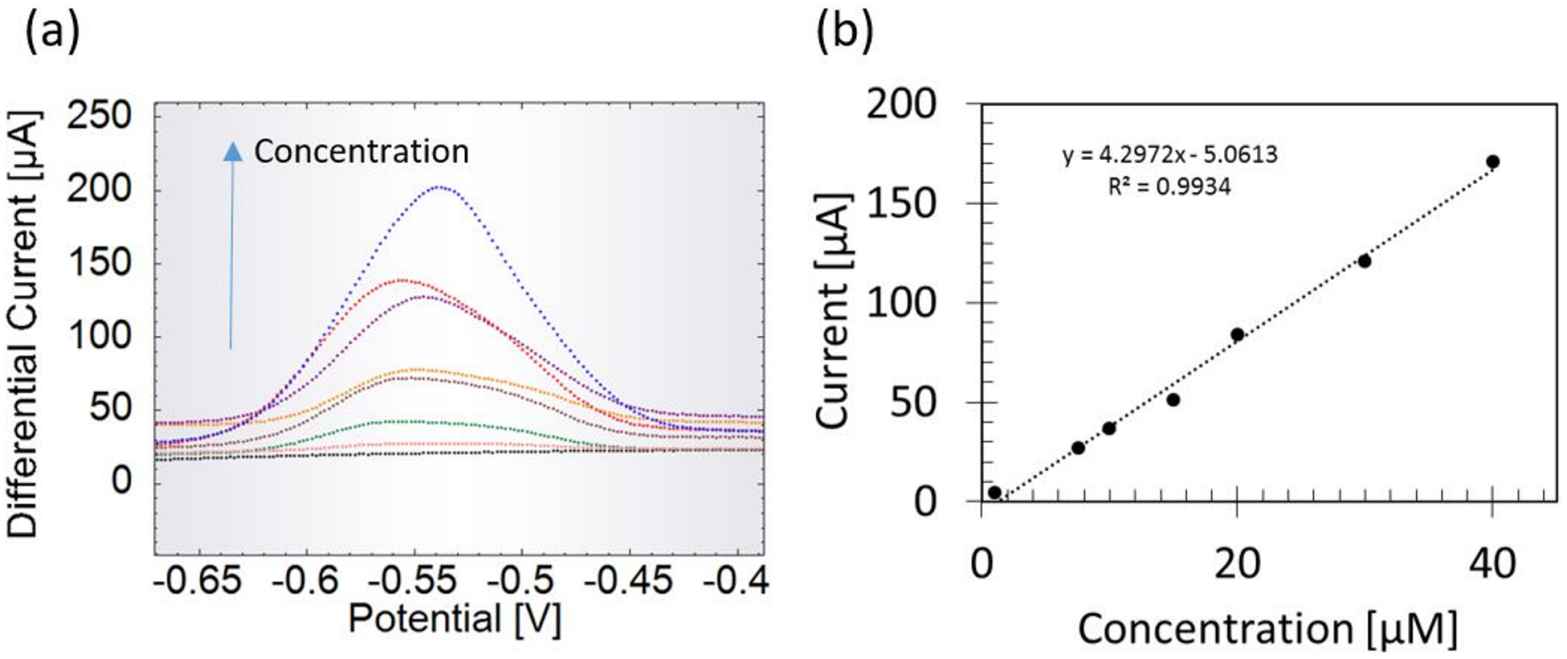
Pyocyanin quantification. Detection of pyocyanin in lysogeny broth (LB) using the paper-based sensors. (a) Square wave voltammograms of increasing pyocyanin concentrations from 1–40 μM in LB. (b) Standard curve using the extracted peak currents as function of the pyocyanin concentration.

The experimental setup is as earlier shown in Alatraktchi et al [13]. The paper-based sensors were used to detect pyocyanin at SWV scans from -0.7 V to -0.3 V. Fig 3a shows that no peak was detected when measuring the LB control (black graph). Square wave voltammograms of spiked LB shows increasing peak heights with increasing pyocyanin concentration. The pyocyanin peak appeared at -0.55 V. To filter out the non-faradaic current from the peak currents, only the peak heights were extracted from the measurements. The extracted faradaic currents were then plotted as function of the corresponding concentrations as seen in (Fig 3b). The R^2^ value of the fitted line is 0.993. The limit of detection is theoretically calculated to 95 nM. The limit of quantification is 3.2 μM. The sensitivity of the paper-based sensors is given by the slope of the standard curve which is 4.3 μA/μM. The standard deviation is 0.14 μM in average based on three replicates of each measured concentration. The paper-based sensors can therefore theoretically detect pyocyanin even below the clinically relevant range that is reported to be between 1 and 100 μM[20].

### Pyocyanin detection using paper-based sensors outcompetes commercial sensors

**Fig 4:**
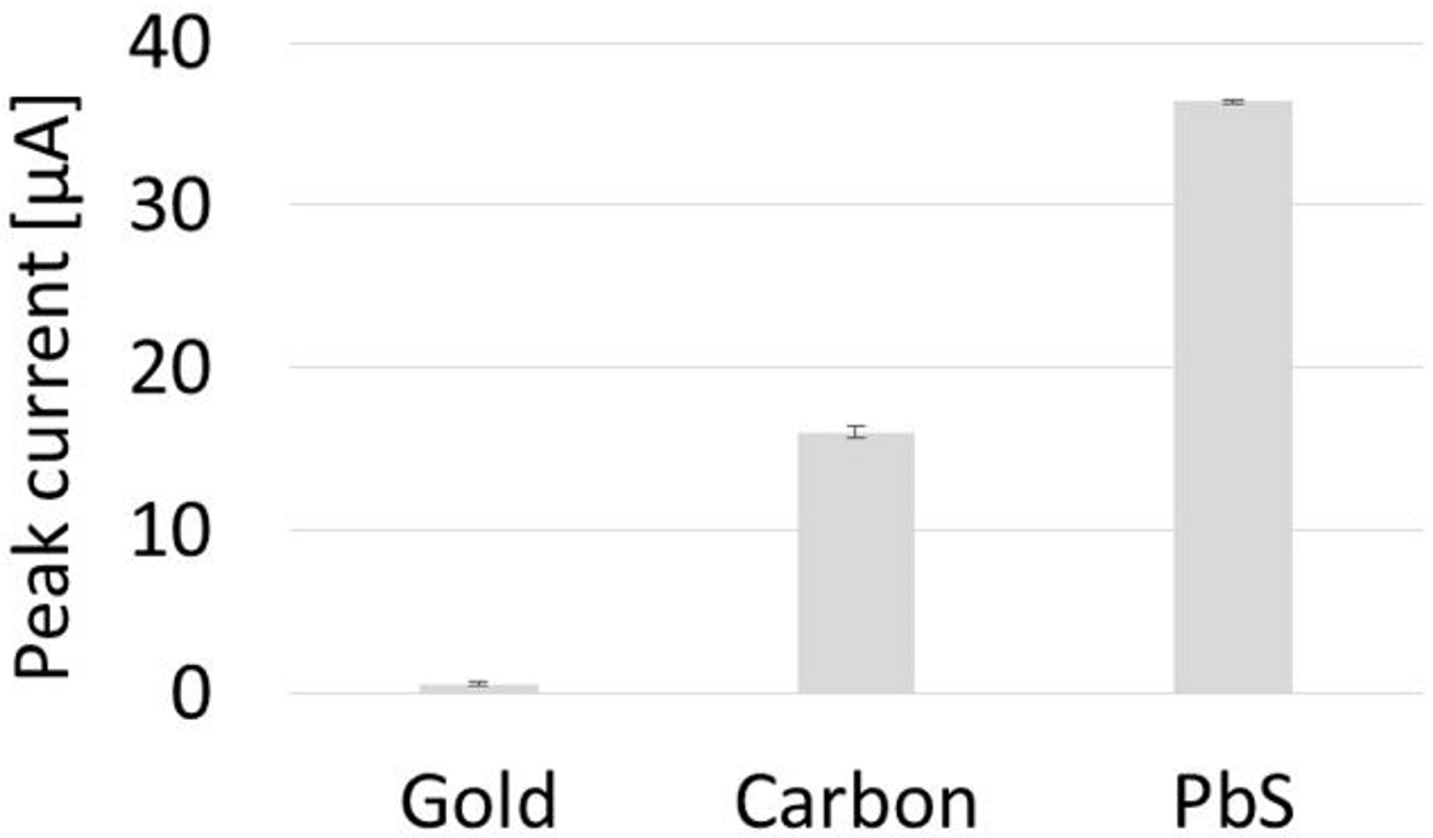
Comparison of sensor performances. Peak currents extracted from square wave voltammograms of 10 μM pyocyanin detected with commercial gold and carbon sensors compared to the new paper-based carbon sensors (PbS).

Commercially available ceramic-based sensors were used as controls for comparison of the paper-based sensors developed in this work. Commercial gold and carbon sensors were respectively used to detect 10 μM pyocyanin (Fig 4). The faradaic currents of the SWVs were extracted by measuring the height of the peaks. As the three working electrodes from the respective sensors have identical surface areas, the peak currents were directly compared. The highest current yield was achieved by the paper-based sensors, followed by the commercial carbon sensor and finally the gold commercial sensor. The accuracy expressed by the average of the standard deviation was best using the paper-based sensors. The paper-based sensors revealed a 2.3 times current yield as the equivalent commercial carbon sensor.

### Detecting pyocyanin release from P. aeruginosa

**Fig 5:**
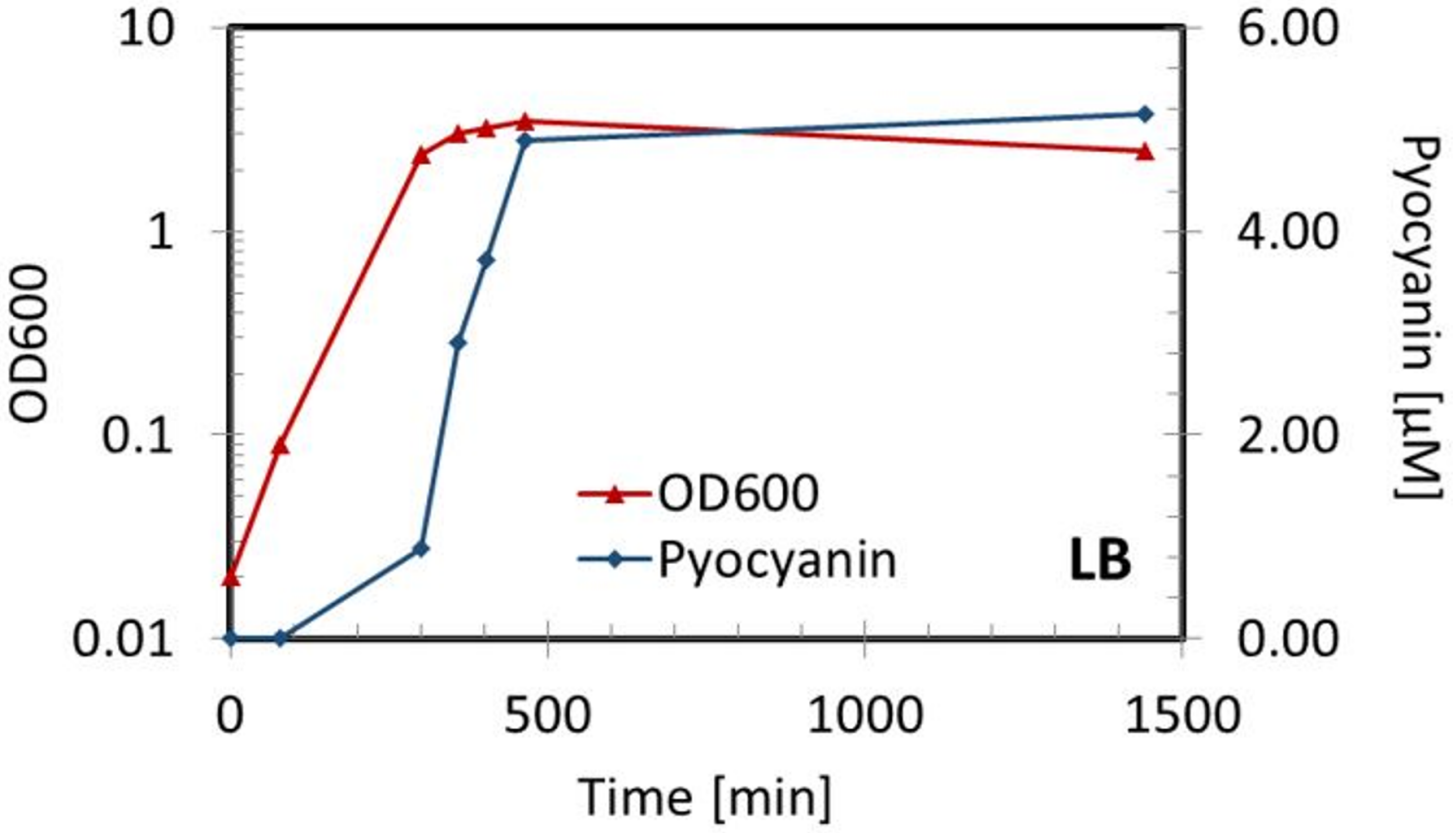
Pyocyanin detection during bacterial growth. Detection of pyocyanin in *P*. *aeruginosa* cultures during exponential and stationary growth in lysogeny broth (LB).

The reference strain *P*. *aeruginosa* (PAO1) was grown into stationary state where the pyocyanin production is at its highest concentration. Samples from the bacterial cultures were collected during exponential and stationary phase. The OD600 and the pyocyanin of all the cultures were measured in triplicates. Fig 5 shows that no pyocyanin was initially produced in the culture. This is expected as it has been illustrated that pyocyanin release is controlled by the quorum sensing system which is not present in the early state of a growing culture[21]. When the bacteria reached early stationary phase the pyocyanin concentration started increasing linearly, reaching maximum concentration of XX μM after 24 hours of growth. The maximum pyocyanin that can be released from *P*. *aeruginosa* cultures depend on several factors such as the nutrient availability, the oxygenation of the culture and the temperature. Common for all cases is the necessity of reaching stationary phase in order to achieve the maximum pyocyanin production threshold of the given strain. These parameters could be relevant to optimize to achieve maximum efficacy and effectiveness in pyocyanin detection in future analysis of clinical strains.

*P*. *aeruginosa* produce several redox active chemicals of most predominant are 2-heptyl-3-hydroxy-4-quinolone (PQS) and 2-heptyl-4-hydroxyquinoline (HHQ). However, these chemicals are redox active at different potentials and their respective signals therefore do not overlap electrochemically. The electrochemical signal of pyocyanin appears at a unique potential that lies outside the interference window of the other redox-active chemicals, which enables selective detection without interference as has been shown by Alatraktchi et al. and Buzid et al. [13,14,22]. Future studies must address the contribution of interfering molecules secreted by other microorganisms in order to achieve selective detection of pyocyanin in clinical samples.

## Conclusion

The paper-based sensor presented here demonstrates that it is possible to achieve precise, rapid and sensitive measurements of pyocyanin directly produced by *P*. *aeruginosa* using low-cost disposable electrochemical paper-sensors. The projected capital equipment cost for the potentiostat and the possibility of in-house fabrication of disposable paper-based sensors make this an attractive system for rapid screenings of bacterial cultures in low resource settings. Pyocyanin can instantly be quantified in samples without any pretreatment of the sample, which has until now included risky chloroform based extractions or complicated equipment settings. Future development of the paper-based sensors might allow the use of these sensors for point-of-care diagnostics of *P*. *aeruginosa* directly on patient samples such as sputum from cystic fibrosis patients or patients with pneumonia, in the urine of patients with urinary tract infection or in the blood from patients with bacteremia.

## Supporting information

S1 File Experimental data. Experimental data on the calibration, sensor comparison and PAO1 growth.
(XLSX)
